# miR-216a-targeting theranostic nanoparticles promote proliferation of insulin-secreting cells in type 1 diabetes animal model

**DOI:** 10.1038/s41598-020-62269-4

**Published:** 2020-03-24

**Authors:** Ping Wang, Qiong Liu, Hongwei Zhao, Jack Owen Bishop, Guoli Zhou, L. Karl Olson, Anna Moore

**Affiliations:** 1 Precision Health Program, Department of Radiology, College of Human Medicine, Michigan State University, East Lansing, Michigan 48823 USA; 2 Department of Anatomy, Histology and Embryology, School of Basic Medical Science, Fudan University, Shanghai, 200032 China; 3 Shanxi Medical University, Taiyuan, Shanxi 030001 China; 4 Department of Gynecologic Oncology, Shanxi Provincial Cancer Hospital, Taiyuan, Shanxi 030013 China; 5 Department of Neuroscience, College of Natural Science, Michigan State University, East Lansing, Michigan 48824 USA; 6 Biomedical Research Informatics Core, Clinical & Translational Sciences Institute, Michigan State University, East Lansing, Michigan 48824 USA; 7 Department of Physiology, College of Natural Science, Michigan State University, East Lansing, Michigan 48824 USA

**Keywords:** Endocrine system and metabolic diseases, Endocrinology, Biomarkers, Cell delivery, Oligo delivery

## Abstract

Aberrant expression of miRNAs in pancreatic islets is closely related to the development of type 1 diabetes (T1D). The aim of this study was to identify key miRNAs dysregulated in pancreatic islets during T1D progression and to develop a theranostic approach to modify their expression using an MRI-based nanodrug consisting of iron oxide nanoparticles conjugated to miRNA-targeting oligonucleotides in a mouse model of T1D. Isolated pancreatic islets were derived from NOD mice of three distinct age groups (3, 8 and 18-week-old). Total RNA collected from cultured islets was purified and global miRNA profiling was performed with 3D-Gene global miRNA microarray mouse chips encompassing all mouse miRNAs available on the Sanger miRBase V16. Of the miRNAs that were found to be differentially expressed across three age groups, we identified one candidate (miR-216a) implicated in beta cell proliferation for subsequent validation by RT-PCR. Alterations in miR-216a expression within pancreatic beta cells were also examined using *in situ* hybridization on the frozen pancreatic sections. For *in vitro* studies, miR-216a mimics/inhibitors were conjugated to iron oxide nanoparticles and incubated with beta cell line, βTC-6. Cell proliferation marker Ki67 was evaluated. Expression of the phosphatase and tensin homolog (PTEN), which is one of the direct targets of miR-216a, was analyzed using western blot. For *in vivo* study, the miR-216a mimics/inhibitors conjugated to the nanoparticles were injected into 12-week-old female diabetic Balb/c mice via pancreatic duct. The delivery of the nanodrug was monitored by *in vivo* MRI. Blood glucose of the treated mice was monitored post injection. *Ex vivo* histological analysis of the pancreatic sections included staining for insulin, PTEN and Ki67. miRNA microarray demonstrated that the expression of miR-216a in the islets from NOD mice significantly changed during T1D progression. *In vitro* studies showed that treatment with a miR-216a inhibitor nanodrug suppressed proliferation of beta cells and increased the expression of PTEN, a miR-216a target. In contrast, introduction of a mimic nanodrug decreased PTEN expression and increased beta cell proliferation. Animals treated *in vivo* with a mimic nanodrug had higher insulin-producing functionality compared to controls. These observations were in line with downregulation of PTEN and increase in beta cell proliferation in that group. Our studies demonstrated that miR-216a could serve as a potential therapeutic target for the treatment of diabetes. miR-216a-targeting theranostic nanodrugs served as exploratory tools to define functionality of this miRNA in conjunction with *in vivo* MR imaging.

## Introduction

Theranostic nanomedicine is a novel interdisciplinary approach that integrates nanotechnology, biomedicine and imaging^1–4^. Theranostic nanodrugs can deliver therapeutics including small molecules, peptides, antibodies and oligonuceotides to targeted tissues, while imaging allows the non-invasive assessment of their biodistribution and pharmacokinetics^5–7^. To date, several nanomedicine drug delivery systems based on this theranostic concept have been translated into clinical trials including applications in oncology, infection and inflammation^8^.

Loss of beta cell mass and function underlies the pathology of both type 1 and type 2 diabetes^9^. As pancreatic beta cells play a central role in the disease progression, anti-diabetic therapies should focus on the ways to improve glucose homeostasis by preserving, expanding and/or improving the function of this key cell type^10^. One of the therapeutic approaches that has recently gained a significant interest deals with targeting microRNAs (miRNA) - small, non-coding ribonucleotides that function as negative regulators of gene expression^11^. These regulatory molecules play major roles in normal cellular function as well as in a wide variety of pathological conditions including cancer, inflammation, cardiovascular disease and viral infections^12^. There is an overwhelming evidence that miRNAs also play an important role in the regulation of glucose homeostasis and proper beta cell function and thereby may contribute to diabetes development^13,14^. Although a number of miRNAs from pancreatic beta cells have been identified using various screens^15^, functional studies that link most of the identified miRNAs to regulation of beta cell function remain unknown.

**Figure 1:**
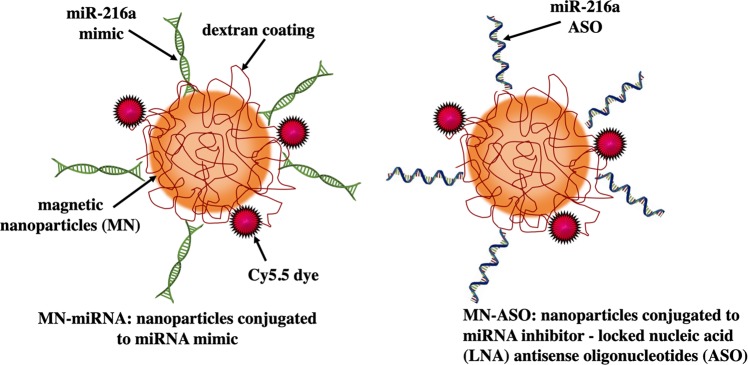
Schematic representation of dextran‐coated magnetic nanoparticles conjugated with the near infrared fluorescent dye Cy5.5 and miR-216a mimic or inhibitor.

Identification of key miRNAs involved in beta cell proliferation could help design novel theranostic strategies based on modulation of cellular miRNA profile and expanding the pool of viable beta cells. In this study, we found that microRNA-216a (miR-216a) was upregulated during diabetes progression and played a role in the acquisition of a phenotype of proliferating beta cells. To this end, we performed gain/loss of function studies to elucidate miR-216a role in diabetes development using the engineered image-guided nanodrug. The theranostic nanodrug consisted of magnetic nanoparticles (MN) used for delivery of miRNA mimics (MN-miRNA) or inhibiting locked nucleic acid (LNA) antisense oligonucleotides (ASO) (MN-ASO) to beta cells (Fig. 1). Our studies demonstrated that delivery of miRNA mimic nanodrug to pancreatic islets monitored by MRI resulted in increased insulin secretion and beta cell proliferation. In summary, in conjunction with *in vivo* imaging, miR-216a could serve as a potential therapeutic target for the treatment of diabetes.

## Materials and Methods

### Animals and islet isolation

All animal experiments were performed in compliance with the National Institutes of Health guide for the care and use of Laboratory animals (NIH Publications No. 8023, revised 1978) and approved by the Institutional Animal Care and Use Committee at Michigan State University. For miRNA profiling study, female NOD/ShiLtJ mice (The Jackson Laboratory, Bar Harbor, ME) were used, as their diabetes incidence rate is significantly higher than in males^16^. Animals with two consecutive blood glucose readings of >250 mg/dl were considered diabetic.

Islets were isolated from the pancreata of 3-week-old (early pre-diabetic, no insulitis), 8-week-old (pre-diabetic, insulitis being initiated), and 18-week-old (late pre-diabetic stage, late stage insulitis, and confirmed diabetes) NOD mice by collagenase digestion as described in^17^. Isolated islets were cultured for over 16 hours to allow for the escape of islet-infiltrating lymphocytes^15,18^.

### Total RNA isolation and miRNA profiling microarray

Total RNA containing miRNAs was isolated from the three groups (n = 9 mice/group) using miRNeasy Mini Kit (Qiagen, Valencia, CA). Global miRNA profiling was performed using Toray’s 3D-Gene miRNA oligo chip v.16 (Toray Industries, Tokyo, Japan)^19–21^. This array includes the analysis of the murine miRNA available on miRBase (V16). All microarray experiments were performed in duplicate. The chips were stringently washed after incubation with RNA samples, and fluorescence signals were scanned with a 3D-Gene Scanner 3000 and analyzed using 3D-Gene Extraction software. The expression levels of each miRNA were globally normalized using the background-subtracted signal intensity of the entire miRNAs in each microarray. Hybridized probe spots with signal intensity greater than the mean intensity plus 2 standard deviations of the background signal were considered to be significant. All data obtained from the microarray experiments were normalized by a quantile normalization method^22^, and then filtered (75 percentile of miR expression >6 in log2 scale)^20^. Principal components analysis (PCA) was performed using GenEx Enterprise software. This analysis was used to reduce the dimensionality of multivariate data into a multi-dimensional space, allowing for clear visualization of the variation between different samples types^23^, which enables a biological interpretation of the nature of coherent variation^24^.

### Candidate miRNA selection, verification and analysis of downstream targets of selected miRNA

The candidate miRNAs and downstream targets linked to cell proliferation were assessed using Ingenuity Pathway Analysis (IPA, Qiagen Bioinformatics, Frederick, MD)^25^. IPA incorporates experimentally demonstrated and predicted miRNA-mRNA interactions from the TarBase, miRecords, and TargetScan databases, as well as from peer-reviewed miRNA original research articles as the content base for the miRNA Target Filter (http://www.ingenuity.com/products/ipa/microrna-research). miRNAs whose predicted mRNA targets are linked to aspects of proliferation were selected for further analysis by RT-qPCR and *in situ* hybridization (ISH). Four mature miRNAs (miR-216a, miR-29b, miR-375 and miR-7) identified by miRNA microarray were assessed by RT-qPCR using the miScript II RT kit (Qiagen, Frederick, MD). Total RNA was extracted from islets isolated from NOD mice of three different ages using RNAspin Mini Isolation Kit (GE Healthcare, Chicago, IL), and cDNAs of total mature miRNA were synthesized using miScript II RT Kit (Qiagen, Frederick, MD). RT-qPCR was performed using Applied Biosystems ABI PRISM 7500 Sequence Detection System (Life Technologies Corporation, Carlsbad, CA). Primers were purchased from Qiagen. Endogenous 18 S ribosomal RNA was used as an internal control for normalizing gene expression. All samples were run in triplicate. ISH analysis was performed using the miRCURY LNA miRNA detection probes (Exiqon, Denmark) designed with optimal LNA positioning to achieve high sequence specificity, low secondary structure and minimal self-annealing. For ISH, pancreatic frozen sections were fixed with 4% paraformaldehyde for 10 mins at room temperature, followed by treatment with 5 μg/ml proteinase K for 5 min at room temperature and pre-hybridization in hybridization buffer for 4 hours. After denaturation of miR-216a probes, the sections were incubated with miR-216a probes in hybridization buffer (2.5 pmol/150 μl) at 58 °C overnight in a humidified chamber. After washing with pre-warmed SSC buffer, the sections were examined under the microscope^26^. Expression of the downstream targets was assessed by immunostaining on pancreatic sections from NOD mice. For localizing the signal from the miRNA downstream targets in insulin expressing cells, we performed ISH and immunostaining on consecutive pancreatic tissues sections of the pre-diabetic and diabetic NOD mice. Consecutive sections were double stained for insulin/CD3, PTEN/CD3, Ki67/CD3 and insulin/Ki67^27^.

### Synthesis and characterization of miR-216a nanodrugs

The nanodrugs consisted of magnetic nanoparticles (MN, magnetic resonance imaging moiety) conjugated to LNA ASO or miRNA mimics (Exiqon, Denmark) as a therapeutic moiety (Fig. 1). The sequences of miRNA mimics, inhibitors and scramble controls used for the synthesis of the nanodrugs are included in Supplemental Table 1. Nanoparticles with a size of ~30 nm were used for conjugation to the oligonucleotides^28^. Synthesis of the prototype nanodrugs have been previously described by us^6,29^ and involved: (1) synthesis of dextran-coated magnetic nanoparticles^30^; (2) conjugation of Cy5.5 fluorescent dye. The number of dyes per magnetic nanoparticle was determined as 3.2 (6); (3) conjugation of antisense LNA inhibiting oligonucleotides (ASO) or miRNA mimics through heterobifunctional linker N-succinimidyl 3-[2-pyridyldithio]-propionate (SPDP; Thermo Scientific Co., Rockford, IL) to produce MN-ASO or MN-miRNA^31–33^. ASO-scrambled (MN-ASOscr, single stranded) and mimics-scrambled (MN-miRNAscr, double stranded) nanodrugs were also synthesized as controls. The number of oligos per MN was determined as 4.0 using the electrophoresis analysis method described previously^33^.

### Evaluation of miRNA targets in a beta cell line after treatment with the nanodrugs *in vitro*

Murine insulinoma beta cell line beta-TC6 (American Type Culture Collection, Manassas, VA), was used for *in vitro* experiments. Cells were incubated with MN-ASO or MN-ASOscr nanodrugs (25 μg iron/ml) for 48 hrs. Separately, four groups of cells were incubated with MN-miRNA or MN-miRNAscr (25 μg iron/ml) nanodrugs for 48 hrs in the medium with or without 10% FBS. The same incubations were performed with the islets isolated from Balb/C mice. miR-216a expression levels in beta-TC6 cells after incubation with the nanodrugs were assessed by RT-qPCR using the miScript II RT kit. Endogenous 18 S ribosomal RNA was used as an internal control. Glucose-stimulated insulin secretion was evaluated using static incubation of beta-TC6 cells treated with the nanodrugs at low (1.7 mmol/L) and high (20 mmol/L) glucose concentrations. Insulin was measured in supernatants and cell extracts using an ELISA kit (Mercodia, Uppsala, Sweden). Glucose stimulation index was calculated as the ratio of stimulated to basal insulin secretion normalized by the insulin content. Nanoparticle toxicity was determined by assessing cellular viability using colorimetric (3-(4,5- dimethylthiazol-2-yl)-2,5-diphenyltetrazolium bromide assay (MTT, Promega, Madison, WI). Western blot analysis was performed as described previously^6,34^. Protein extracts from beta-TC6 cell line as well as from the islets from Balb/C mice were subjected to electrophoresis on 10% SDS-polyacrylamide gels. The membrane was then incubated with anti-PTEN antibodies (1:500, Abcam, Cambridge, MA) followed by the respective secondary antibodies conjugated to horseradish peroxidase. The antibody-reactive bands were identified by enhanced chemiluminescence reagents (GE Healthcare, Chicago, IL), and exposed and imaged with ChemiDoc MP Imaging System (Bio-Rad Laboratories, Hercules, CA). Western blot results were quantified using an ImageJ 1.46r software (NIH).

For immunocytochemistry, beta-TC6 cells were incubated with primary anti-PTEN (1:100, Abcam, Cambridge, MA) and anti-Ki67 (1:100, Abcam, Cambridge, MA) antibodies followed by incubation with fluorescently labeled secondary antibody. Slides were mounted with a mounting medium containing DAPI (Vectashield, Vector Laboratories, Inc., Burlingame, CA). Images were acquired on a Nikon Eclipse 50i microscope using a SPOT 7.4 Slider RTKE CCD camera (Diagnostic Instruments, Sterling Heights, MI), analyzed with iVision 4.015 software^34^ and quantified using an ImageJ 1.46r software (NIH) according to^35^.

### Intrapancreatic ductal injection of the nanodrugs and assessment of their delivery by *in vivo* MRI

Diabetes was induced in 12‐week old female BALB/c mice (four groups, n = 5/group, Jackson Laboratories, Bar Harbor, ME) by intraperitoneal injection of streptozotocin (STZ, Sigma‐Aldrich, St. Louis, MO, three consecutive injections, 120 mg/kg) freshly dissolved in sodium citrate buffer (pH 7.2)^36^. Mice were considered diabetic if blood glucose readings reached above 250 mg/dL on two consecutive measurements. Nanodrugs (MN-ASO, MN-ASOscr, MN-miRNA and MN-miRNAscr, 15 mg iron/kg) were injected into the diabetic animals after a laparotomy and exposure of the duodenum and the pancreas using a 31‐gauge blunt-ended infusion catheter (World Precision Instruments, Sarasota, FL) inserted in the pancreatic duct through the sphincter of Oddi^28^. The catheter was connected to a quintessential stereotaxic injector (Stoelting, Wood Dale, IL) to allow for a stable infusion of the nanoparticles over a 15 min period^28^. The process was monitored by *in vivo* MR imaging using a 9.4 T Bruker scanner equipped with a Rat Array MRI CryoProbes coil and using standard T2 weighted gradient echo pulse sequences and T2 maps. Imaging was performed before and 3 days after the nanodrug injection. Images were analyzed as previously described^6,28^.

### Evaluation of therapeutic efficacy of the nanodrugs in a type 1 diabetes animal model

Therapeutic studies were performed in STZ-injected BALB/c mice. Four group of mice were injected with MN-ASO, MN-ASOscr, MN-miRNA or MN-miRNAscr (15 mg iron/kg). To assess the effect of the nanodrugs on reaction to glucose stimulus, we performed Intraperitoneal Glucose Tolerance Test (IPGTT) 7 days post the nanodrug injection. After fasting overnight mice received an intraperitoneal injection of a 25% glucose (2 g glucose/kg). Glucose measurements were taken from blood drawn from a tail snip.

Immunohistochemistry was performed on the frozen pancreatic tissue slices. The slices were incubated with the following primary antibodies: 1:200 guinea pig anti-insulin (Abcam, Cambridge, MA), 1:100 rabbit anti-PTEN (Abcam, Cambridge, MA), and 1:100 rabbit anti-Ki67 (Abcam, Cambridge, MA), followed by the staining with fluorescently labeled secondary antibodies. Cell nuclei were visualized with DAPI. Images were collected using a Nikon fluorescence microscope and semi-quantitatively analyzed using an ImageJ 1.46r software. Briefly multi-color fluorescent images were split into single channels and converted to grayscale images. The area of interests (damaged islets) was selected using freehand selection tool in ImageJ 1.46r software (NIH). The Corrected Total Cell Fluorescence (CTCF, the unit of measurement utilized by ImageJ to quantify specific levels of fluorescence) was calculated using the following formula: CTCF = Integrated Density − (Area of selected cell × Mean fluorescence of background readings).

### Examination of *in vivo* systemic toxicity of the nanodrugs in type 1 diabetes mouse model

Seven days after the nanodrugs injection, animals were scarified, their blood was collected, and serum was separated by centrifugation. Serum chemistry including liver and kidney function and blood indicators of pancreatitis was performed by Catalyst Dx Chemistry Analyzer (IDEXX Laboratories, Westbrook, ME).

### Statistical analysis

All experiments performed in duplicate or triplicate were repeated using independent samples. Comparison between miRNA profiles from the three groups was performed using Analysis of Variance (ANOVA) and post hoc pairwise analysis with Student t-tests. To control for the false discovery rate (FDR), we used the Benjamini-Hochberg method with the R p. adjust function to compute adjusted p values (FDR). The miRNAs from microarray analysis with p < 0.05, FDR < 0.10, and fold change ≥ 2 were considered statistically significant. miRNA microarray analysis was also performed using the PCA^24^. Pearson correlation coefficient analysis was performed between miR216a levels and infiltrated CD3 + T cells. For other non-microarray experiments, differences between the time points and between experimental and control groups were assessed by a Student t test and corrected by the ANOVA (GraphPad Software); the repeated two-way ANOVA was used for the time course analysis; a p value ≤ 0.05 was considered statistically significant.

## Results

### Profiling of miRNA expression in islet cells

**Figure 2:**
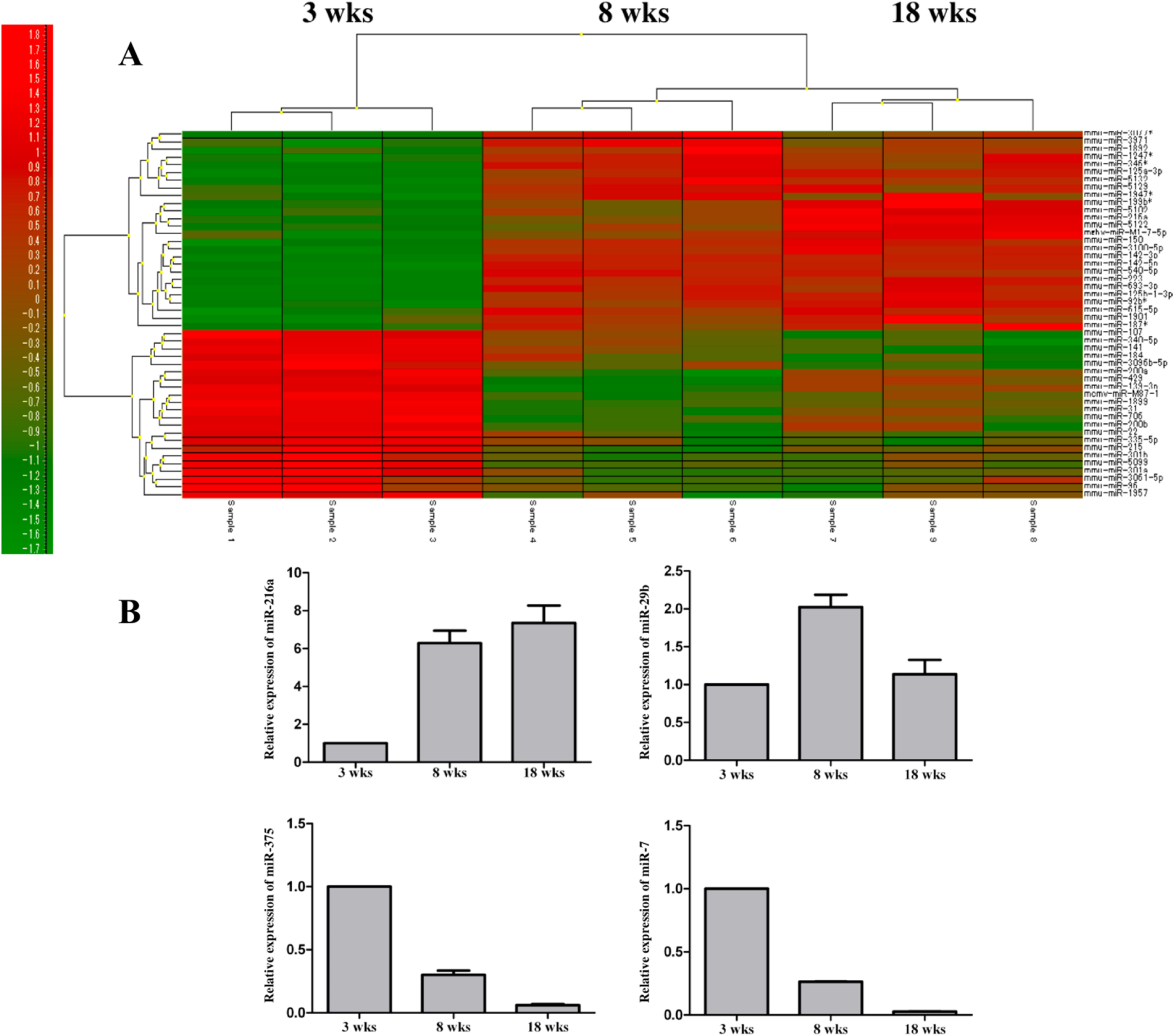
miRNA profile of pancreatic islets from NOD mice. (**A**) A heatmap of miRNA expression in pancreatic islets from 3-, 8- and 18-week old NOD mice (n = 9). Red and green represent high and low microRNA expression; (**B**) miRNA216a, 29b, 375, and 7 expression by real-time quantitative RT-PCR in pancreatic islets from 3-, 8- and 18-week old NOD mice. All experiments were performed in triplicate.

Microarray analysis of miRNAs expression in the islets from 3-, 8-, and 18-week-old female NOD mice revealed that the development of diabetes is associated with the regulation of 1,074 miRNAs (fold change ≥2.0, p-value <0.05, and FDR < 0.10, Supplemental Table 2). As shown in Fig. 2A, all miRNAs considered further in these studies exhibited differential expressions in the miRNA microarray heatmap. PCA results among three sample groups are shown in Supplemental Fig. 1. Microarray experiments were performed in triplicate.

Using an independent set of islets from 3-, 8-, and 18-week-old NOD mice, we performed qPCR analysis of selected miRNAs including miRNA-216a, miRNA-29b, miRNA-375, and miRNA-7, which validated the findings of the miRNA microarray platform (Fig. 2B). These experiments were performed in triplicate. Next, we applied IPA for identifying the miRNA and downstream target for further study. Based on the bioinformatic search, among all of the identified miRNAs, we chose miR-216a as the candidate for further testing since it has been implicated in cell proliferation with PTEN identified as its direct downstream target^37–39^.

### Verification of miR-216a expression on pancreatic sections

**Figure 3:**
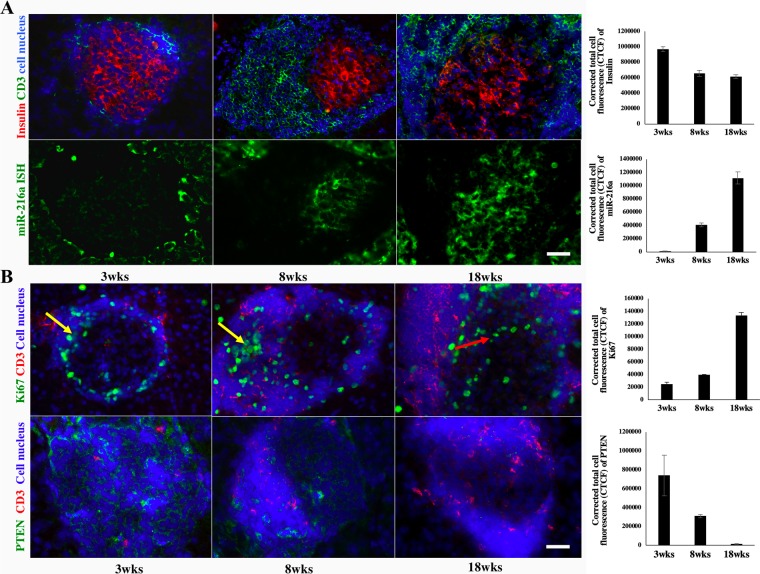
Verification of miR-216 and PTEN expressions in pancreatic islets performed on consecutive sections from NOD mice. All experiments were performed in triplicate. (**A**) *In situ* hybridization analysis of miR-216a expression in pancreatic islets from 3-, 8- and 18-week old NOD mice (bottom, miR-216a: green, magnification bar = 50 μm); Insulin (red) and CD3 (green) immunostaining of consecutive sections (top, cell nucleus – blue). (**B**) Immunostaining of PTEN (green) and CD3 (red) expression in pancreatic islets from 3-, 8- and 18-week old NOD mice (bottom, cell nucleus - blue, magnification bar = 50 μm); Immunostaining of Ki67 (green) and CD3 (red) expression in pancreatic islets from 3-, 8- and 18-week old NOD mice (top, cell nucleus – blue). Note Ki67 positive cells located in the periphery of the damaged islets of 3 and 8 wks old mice (yellow arrows). In contrast, in 18 wks old mice Ki67 positive cells (red arrow) were located in the central area of the damaged islet, where the residual insulin producing cells are located on the consecutive section in (**A**).

ISH and double-immunostaining for miR-216a on consecutive frozen pancreatic sections from 3-, 8-, and 18-week-old NOD mice verified that its expression increased during diabetes development (Fig. 3A). We have performed Pearson correlation coefficient analysis between miR216a levels and infiltrated CD3 + T cells and found significant correlation (r = 0.9403, 95% confidence interval = 0.7355 to 0.9877, p value = 0.0002). We investigated the changes in expression of PTEN, miR-216a target. We found that the upregulation of miR-216a resulted in blocking of PTEN, which is consistent with the previous findings^37–39^. Here, we found that the levels of miR-216a and its target PTEN are inversely correlated throughout the development of diabetes in NOD mice (Fig. 3B). It is well known that PTEN contributes to beta cell proliferation, we also observed that cell proliferation in the damaged islets of 18-week-old NOD mice was increased as demonstrated by the double positive cells stained for insulin and Ki67 (Supplemental Fig. 2). ISH and double-immunostaining experiments were performed in triplicate; for each pancreatic section, three islets were examined. Based on these observations, we selected miR-216a as the target for further *in vitro* testing with the nanodrugs.

### miR-216a: target perturbation studies

**Figure 4:**
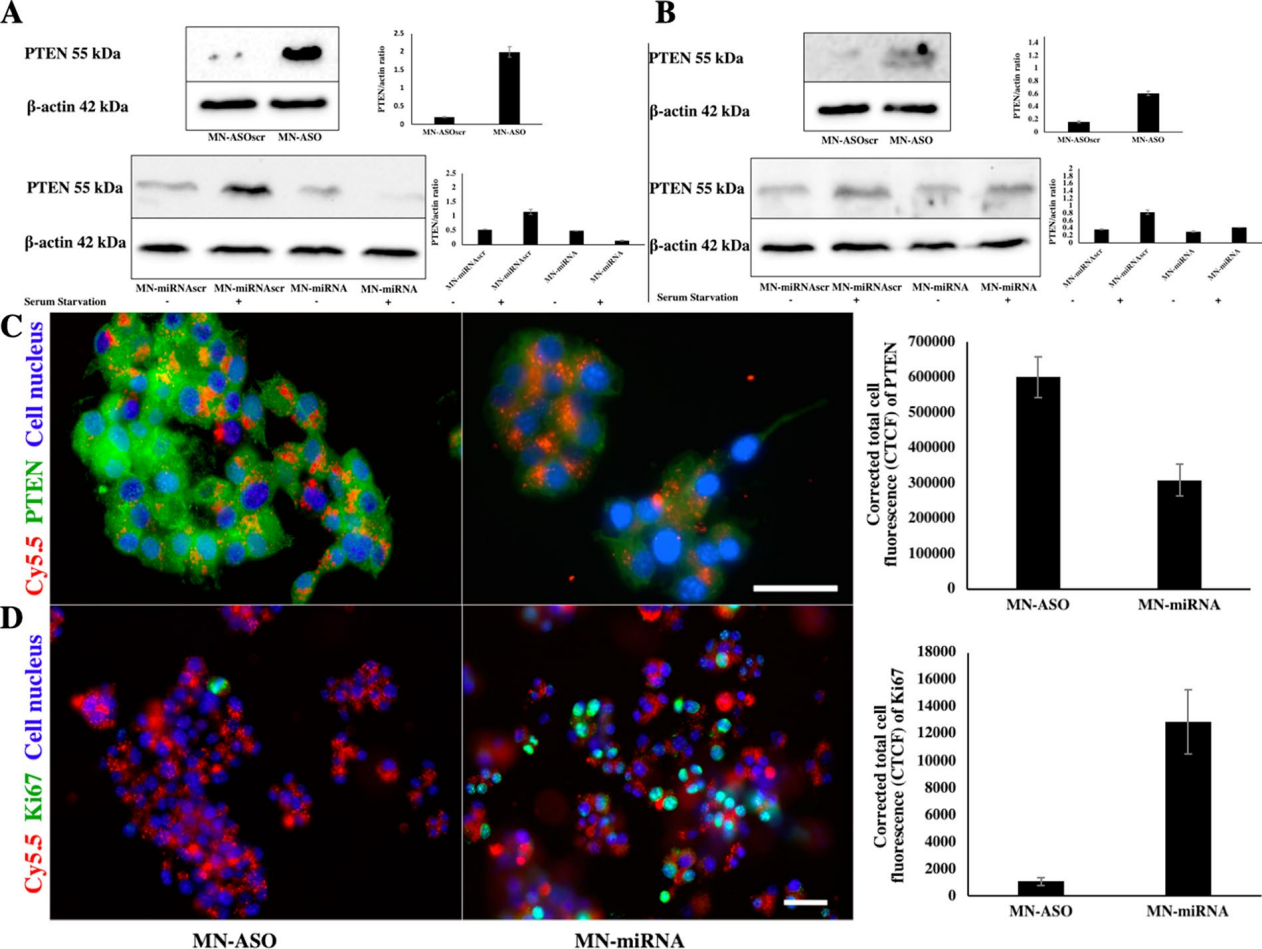
Changes in PTEN expression and cell proliferation caused by miRNA-216 targeting nanodrugs. (**A**) Western blotting analysis of PTEN expression in beta-TC6 cells treated with MN-ASO inhibiting nanodrug or MN-ASOscr nanodrug (top); Note the increase of PTEN expression after treatment with the MN-ASO. Western blot analysis of PTEN expression in beta-TC6 cells treated with MN-miRNA mimic nanodrug or MN-miRNAscr nanodrug (bottom), PTEN expression was compared in beta-TC6 cells incubated with or without FBS (serum starvation condition). Elevated PTEN expression in beta-TC6 cells under serum starvation (SS) was significantly suppressed after treatment with the miR216a mimic nanodrug. All experiments were performed in duplicate. (**B**) Western blot analysis of PETN expression in islets isolated from BALB/c and treated with MN-ASO inhibiting and MN-miRNA mimic nanodrug. All experiments were performed in duplicate. For western blot results, cropped blots are displayed, uncropped blots are included in the Supplemental Information File. beta-TC6 cells (not serum starved) were treated with either MN-ASO inhibiting nanodrug (left) or MN-miRNA mimic nanodrug (right, magnification bar = 25 μm). Note downregulation of PTEN after treatment with the mimic nanodrug. All experiments were performed in triplicate; (**D**) beta-TC6 cells were treated with either MN-ASO inhibiting nanodrug (left) or with MN-miRNA mimic nanodrug (right, magnification bar = 50 μm). Note increased proliferation after treatment with the mimic nanodrug due to downregulation of PTEN, a miR-216a target. All experiments were performed in triplicate.

To investigate the perturbation effects of miR-216a on its target, we first tested miR-216a expression levels in beta-TC6 cells incubated with the nanodrugs and controls. As expected, the MN-ASO significantly inhibited miR-216a, while MN-miRNA notably increased miR-216a expression compared to controls (Supplemental Fig. 3). Glucose-stimulated insulin secretion and glucose stimulation index were not significantly affected in nanodrug-treated groups compared to control (Supplemental Fig. 4A). MTT assay demonstrated that the nanodrugs had no significant effect on cell viability (Supplemental Fig. 4B). We then compared the level of PTEN expression in beta-TC6 cells incubated with miR-216a mimic/inhibitor nanodrugs (Fig. 4A). Western blot analysis showed that in the cells treated with miR-216a inhibitor MN-ASO, PTEN expression was notably increased compared to the cells treated with a scrambled control nanodrug. Western blot analysis also revealed that incubation with miRNA-216a mimic MN-miRNA downregulated PTEN expression level in beta-TC6 cells pre-elevated by serum starvation (Fig. 4A). Western blot analysis of PETN expression in islets isolated from BALB/c mice and treated with either MN-ASO inhibitor or MN-miRNA mimic nanodrugs revealed a similar trend (Fig. 4B). All western blot experiments were performed in duplicate.

Immunostaining also confirmed the Western blot analysis results showing downregulation of PTEN after treatment with miRNA-216a mimic (Fig. 4C). To further confirm whether miR-216a contributed to cell proliferation, we compared the level of proliferation marker Ki67 in beta-TC6 cells incubated with either MN-ASO or MN-miRNA. The results showed that miR-216a mimics increased cell proliferation significantly compared to inhibitor nanodrugs (Fig. 4D).

Cell immunostaining experiments were performed in triplicate.

### Intrapancreatic ductal delivery of the nanodrugs

**Figure 5:**
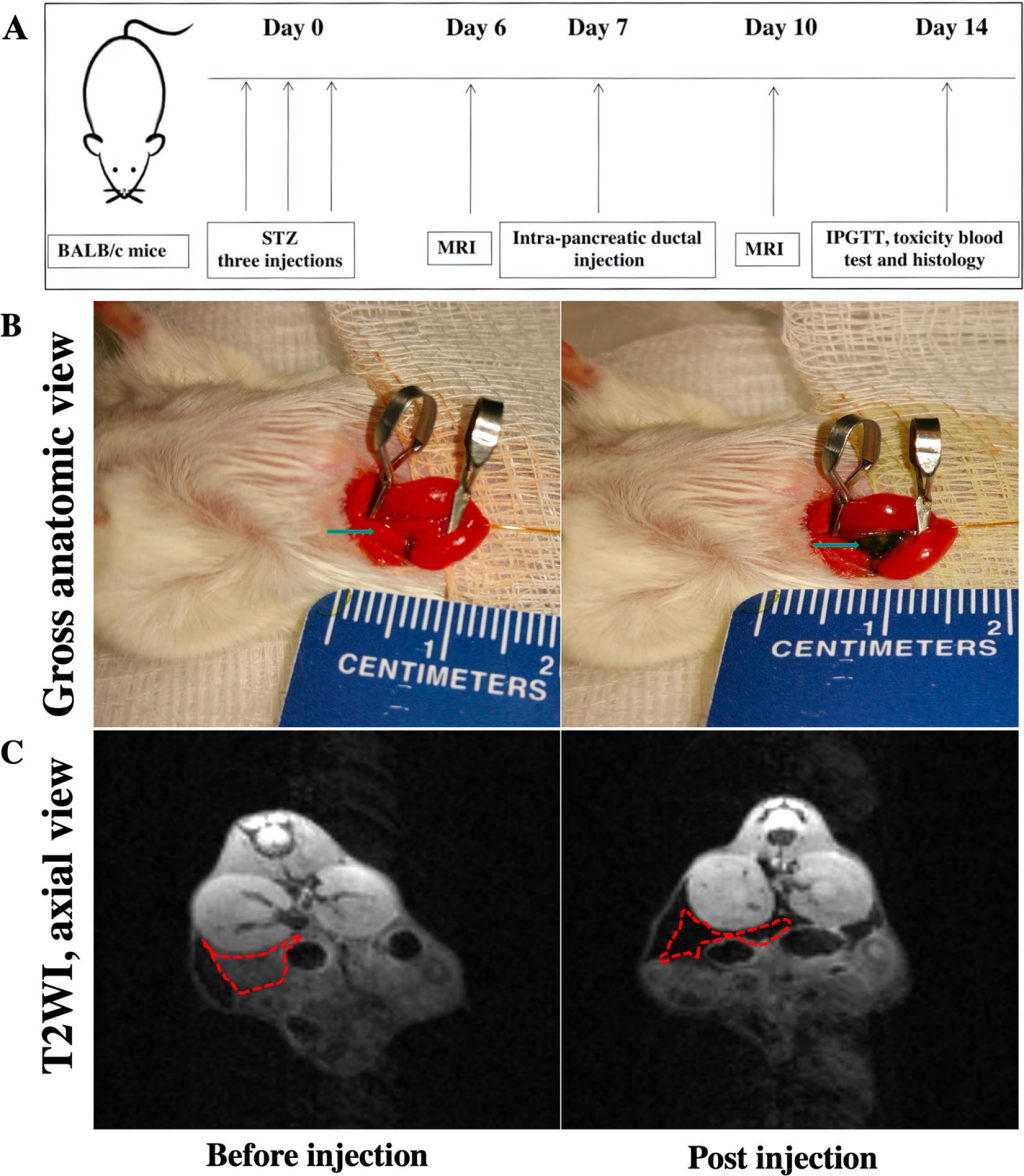
*In vivo* MRI of nanodrugs delivery. (**A**) Experimental flowchart of diabetes induction using streptozotocin (STZ), intra-pancreatic ductal injection of the nanodrugs, magnetic resonance imaging (MRI) and post-injection testing. (**B**) Gross anatomic images of the pancreatic tissue (green arrow) pre‐ and post- intra-pancreatic ductal injection. Note the pancreatic area turning dark post injection, consistent with diffusion of the nanoparticle solution. (**C**) Representative MRI of the nanodrug delivery to the pancreas (MN-miRNA is shown). Axial T2‐weighted images showing loss of signal intensity in the pancreatic tissue (red outline) of diabetic animals 72 hours post injection.

To test whether adjustment of miRNA-216a levels would lead to loss/gain of beta cell proliferation in diabetic animals we performed *in vivo* experiments, in which we treated mice with either MN-ASO or MN-miRNA. To deliver these nanodrugs to the pancreas we utilized intra-pancreatic ductal administration, which we previously used for delivery of parental nanoparticles^28^. To assess accumulation of the nanodrugs in the pancreas, we performed *in vivo* MRI before and 3 days after the injection (Fig. 5A). Visually, pancreatic tissue appeared dark after the injection of all four (two experimental and two control) nanodrugs consistent with magnetic nanoparticle diffusion in the tissue (Fig. 5B, MN-miRNA is shown). Representative T2 map showed a significant decrease in the T2 relaxation time of the post injection pancreas of diabetic animals compared to pre-injection (Fig. 5C, MN-miRNA group, 10.6 ± 2.4 ms vs. 37.2 ± 1.9 ms, p < 0.0001), which confirmed the nanodrug delivery to the pancreatic tissue (Fig. 5B).

### *Ex-vivo* tissue immunostaining

**Figure 6:**
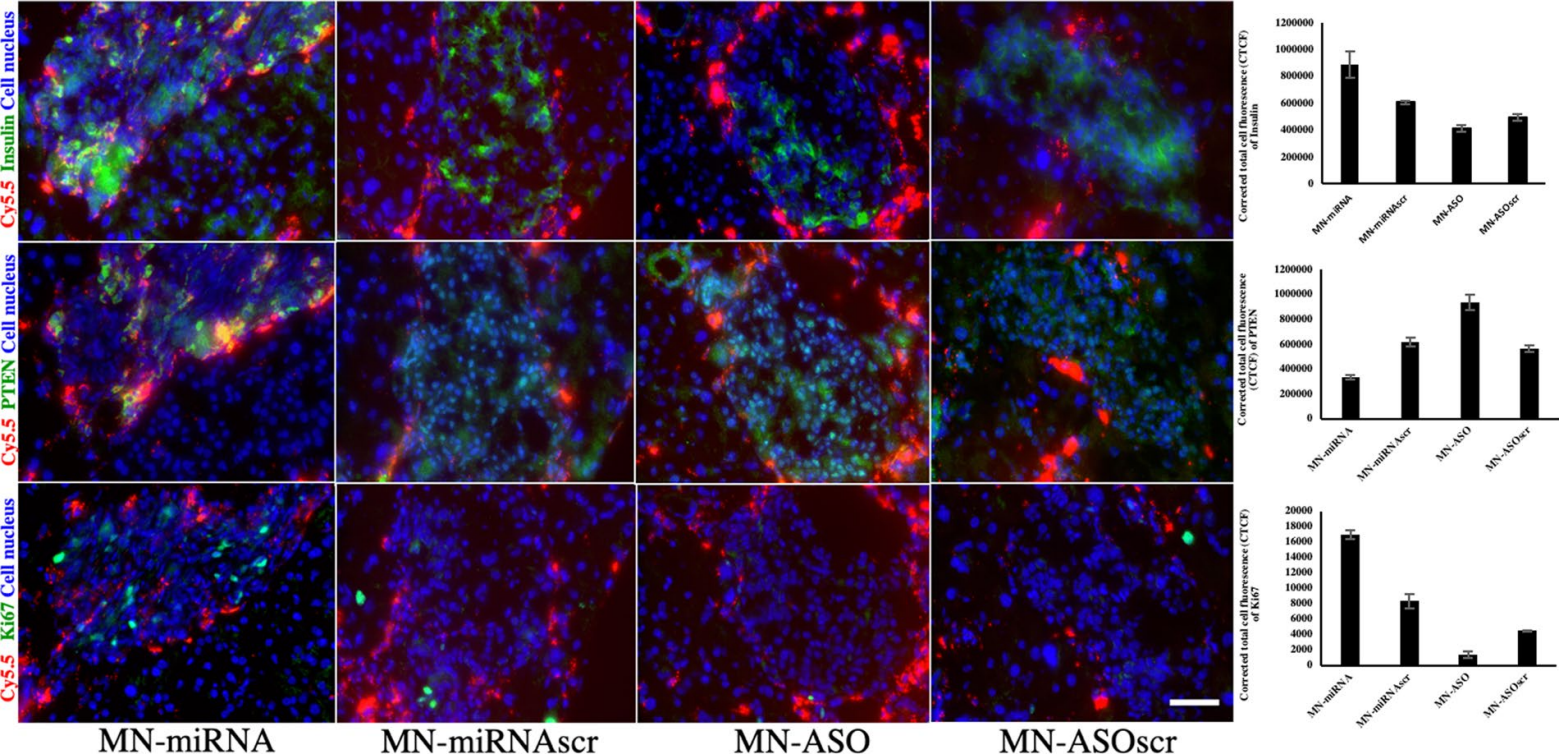
Fluorescence microscopy of consecutive frozen pancreatic sections from STZ-induced diabetic mice injected with MN-miRNA, MN-ASO, MN-miRNAscr and MN-ASOscr. Animals injected with MN-miRNA showed higher insulin expression in pancreatic islets (top: green, insulin; red, Cy5.5; blue, cell nucleus) compared to the animals injected with MN-ASO or control nanodrugs. These animals also showed downregulated PTEN expression in their islets (middle: green, PTEN; red, Cy5.5; blue, cell nucleus) compared to the animals injected with MN-ASO or control nanodrugs. Finally, there was a notably higher cell proliferation in the islets of these animals compared to controls (bottom green, Ki67; red, Cy5.5; blue, cell nucleus); Magnification bar = 40 μm. All experiments were performed in triplicate.

To confirm accumulation of the Cy‐5.5‐labeled nanodrugs in pancreatic islet cells, we performed *ex vivo* histology of pancreatic sections obtained 7 days after intra-pancreatic ductal injection. We observed considerable accumulation of the nanodrugs within pancreatic islets as well as in peripheral area indicated by Cy5.5 signal in the pancreatic sections of STZ injected diabetic animals (Fig. 6). To examine the effect of the nanodrugs on miR-216a target PTEN and proliferation of beta cells, we performed immunostaining for insulin, PTEN, and Ki67 on consecutive pancreatic sections. As shown in Fig. 6, there was a notably lower level of PTEN expression, and higher insulin expression in the islets of the mice injected with MN-miRNA than in those injected with MN-ASO or any of the control nanodrugs. Importantly, cell proliferation was significantly higher in the group injected with MN-miRNA than in the group injected with inhibitor nanodrug MN-ASO where it was virtually absent (Fig. 6). Immunostaining experiments were performed in triplicate; for each pancreatic section, three islets were examined.

### Intraperitoneal glucose tolerance test (IPGTT)

**Figure 7:**
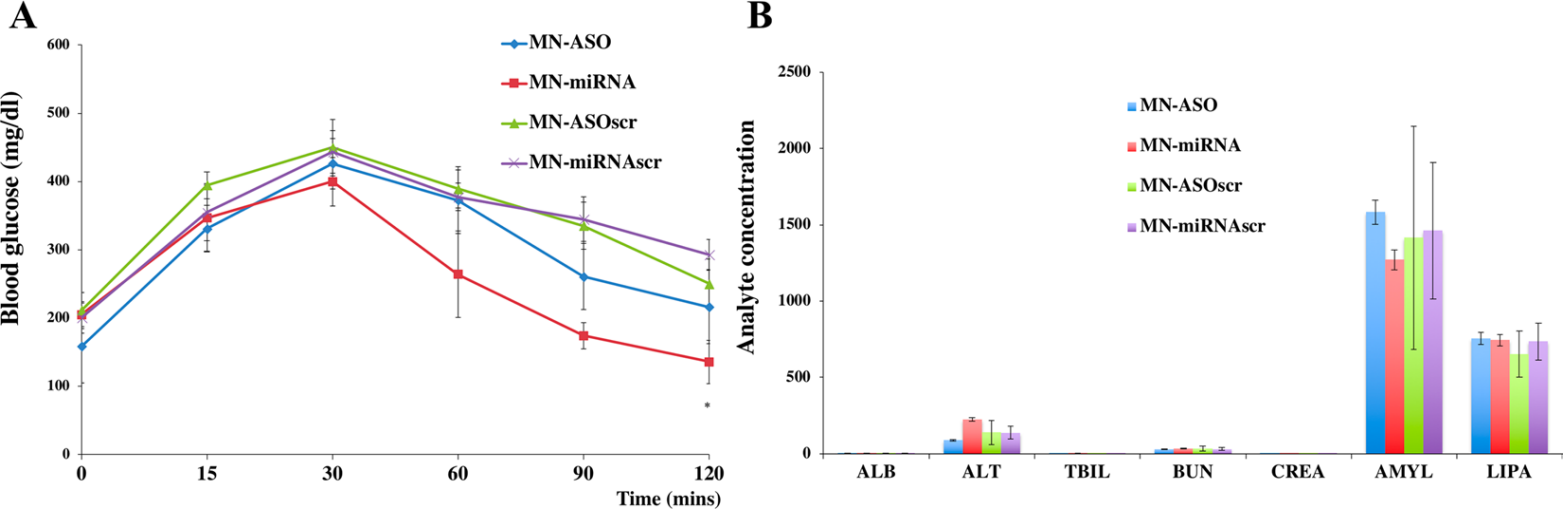
Therapeutic effect of the nanodrugs and toxicity testing in STZ-induced diabetic mice. (**A**) Results of IPGTT in mice injected with MN-ASO, MN-miRNA and controls. There was a significant difference in the glucose disposal curves between the group injected with MN-miRNA and the other three groups. These mice showed significantly lower blood glucose levels at 60 mins after the challenge compared to the other groups (n = 5, *P < 0.05). (**B**) Blood chemistry panel of diabetic animals (n = 5) injected with miR-216-specific nanodrug showed no indication of hepatic, renal or pancreatic toxicity. Albumin (ALB), alanine aminotransferase (ALT), total bilirubin (TBIL), blood urea nitrogen (BUN), creatinine (CREA), amylase (AMYL), lipase (LIPA).

To determine whether the nanodrug injections influenced animals’ ability to respond to a glucose challenge we performed IPGTT. The fasting blood glucose values before and 15 or 30 mins after an intraperitoneal glucose challenge were indistinguishable among four groups (injected with MN-ASO, MN-miRNA, MN-ASOscr or MN-miRNAscr). However, at 60 min after the challenge blood glucose values were significantly lower in the group injected with MN-miRNA (264.2 ± 63.6 mg/dl) compared to control (372.6 ± 48.9 mg/dl) (n = 5, Fig. 7A). These results indicate that the delivery of miR-216a mimic by the nanodrug improved insulin secretion and glucose utilization in diabetic animals.

### Toxicity testing of the nanodrugs

One of the pre-requisites for successful translation of our studies into clinic is the absence of toxicity of the nanodrugs. Therefore, we performed initial toxicity studies that included a focused chemistry panel, including tests for liver and kidney function. We showed that both liver and kidney tests were within the normal range. In addition, blood amylase and lipase tests showed that there was no evidence of pancreatitis after the intra-pancreatic ductal injection, indicating the safety of this procedure (n = 5, Fig. 7B). In summary, our data showed that administration of miR-216 mimic nanodrug resulted in significant beta cell proliferation with no indication of hepatic, renal or pancreatic toxicity.

## Discussion

The discovery of miRNA has generated an enormous research interest to this molecule as a pharmacological target for the treatment of various diseases including diabetes^40,41^. However, there are still challenges that need to be overcome in order to promote miRNAs as a viable therapeutic target. First, miRNA mimics or inhibitors are relatively unstable and chemical modification alters their biological properties. Second, selective targeting of these molecules in insulin-secreting cells has not been established yet. Third, pharmacological over-inhibition or over-expression caused by administration of miRNA mimics or miRNA inhibitors may potentially have profound side effects on beta cell health^41^. Hence, the approach for safe *in vivo* delivery of miRNA mimics or miRNA inhibitors has to be developed.

For miRNA target identification, we compared our miRNA microarray results to the previous studies by Roggli *et al*.^15^ that showed that the expression of miR-29b increased in the islets of 14-week-old NOD mice compared to 4-week-old mice. In our current studies, miR-29b expression was increased in islets from 8-week-old female NOD mice compared to 3-week-old mice. However, the expression of miR-29b was decreased in the islets of 18-week-old female NOD mice as diabetes continued to develop. Roggli *et al*. tested islets from 4, 8 and 14-weeks-old NOD mice with normal blood glucose only. In addition, the authors did not specify the gender of the animals included in the study. However, we know that the diabetes incidence rate is significantly higher in female NOD mice than in males. In our current study, we tested three groups of female mice that included 3, 8 and 18wks old NOD mice with the latter already displaying high blood glucose levels. Therefore, we believe that the animal selection in Roggli *et al*. contributed to the differences between these two studies.

Besides these differences, the miRNA profiling results in our study are quite consistent with those of Roggli *et al*. Both studies found a strong up-regulation of miR-216a in pre-diabetic NOD mice (see Supplementary Material of Roggli *et al*.). Moreover, many other miRNAs strongly up-regulated in Roggli *et al*. including miR-142-3p, miR-142-5p or miR-150 were also found to be upregulated in our study, which strengthens the findings reported here.

To test the hypothesis whether modification of beta cell miRNA profile could promote their proliferation, we chose miR-216a, which has been shown to relate to cell proliferation by a number of investigators^37–39,42–44^. The PCR and ISH data confirmed the miRNA microarray findings that the expression of miR-216a in pancreatic islets increased during the development of T1D, which could be a compensatory mechanism used by the damaged beta cells. We designed and synthesized theranostic nanodrugs that delivered miR-216a-specific mimics/inhibitor to pancreatic beta cells *in vivo*. This delivery was monitored by MRI and resulted in increased insulin secretion and beta cell proliferation when miR-216a mimic nanodrug was used. Importantly, we already observed the effect of the miR-216a mimic after a single injection. We believe that a more dramatic effect could be achieved after multiple injections or by targeting multiple miRNAs related to cell proliferation.

It has been shown that PTEN is a direct downstream target of miR-216a in cancer^38,39^ and kidney disorders^37^. In addition, multiple studies have previously demonstrated that PTEN deletion in beta cells leads to proliferation of these cells *in vivo*^27,45–48^ and protection from STZ-induced diabetes^49^. One study demonstrated that systemic administration of PTEN ASO suppressed PTEN mRNA, and normalized blood glucose in db/db and ob/ob mice^9^. Importantly, studies also showed that PTEN deletion did not lead to tumor formation^50^. Our data suggest that changes in miRNA profile are instrumental in driving the acquisition of beta cell phenotype. Specifically, changes in miR-216 expression are associated with proliferation of beta cells. Inhibition of miR-216a increased the expression of PTEN, resulting in lower cell proliferation whereas the delivery of the miR-216a mimic led to downregulation of its expression and a subsequent increase in the number of proliferating beta cell.

There are several limitations to our study. First, we monitored animals for only one-week post injection because of deteriorating diabetic conditions of the control group animals that did not receive the treatment. After performing these initial studies and seeing positive effect of the nanodrug in the experimental group we will extend our experiments to a longer time course and thoroughly investigate any potential side effects of the nanodrug treatment in addition to blood chemistry described here. Second, to obtain proof-of-principal data we used STZ-induced diabetic model, which is not ideal for observing a genuine autoimmune response. In the future, we plan to test our approach in NOD mice, an accepted model of type 1 diabetes. Finally, in this study, we used non-targeted nanodrugs that accumulate in all types of islet cells based on our previous experience^28^. Therefore, here we are unable to make a claim that proliferation occurs only in beta cells in response to the miR-216a mimic. In the future we will use nanodrugs targeting beta cell-specific markers (such as GLP-1R^29^). In spite of these limitations, there are a few innovative aspects of our study. To the best of our knowledge, this is the first demonstration that delivery of miR-216a mimic *in vivo* suppressed the expression of PTEN, resulting in positive regulation of beta cell proliferation. For the first time we also showed the feasibility of delivery of RNA mimic to beta cells by magnetic nanoparticles and monitor ability to this by *in vivo* MRI. This delivery was performed through intra-pancreatic ductal injection, which could easily be translated into clinic taking into account the existence of an equivalent routine clinical procedure - endoscopic retrograde cholangiopancreatography (ERCP)^51^. Translational potential of our studies is also underscored by the fact that iron oxide nanoparticle that share similar characteristics with the nanoparticles used in our studies have already been approved by the Food and Drug Administration (FDA) for MRI and application of oligonucleotides for therapy has been successfully in clinical trials^52^.

Collectively, our data suggest that the modulation of miR-216a level could be used as a new strategy to promote beta cell proliferation and to protect/replace damaged beta cells. In addition, the described strategy could be potentially used to deliver multiple RNA molecules to alter various aspect of diabetogenesis. The ability to monitor this delivery *in vivo* by MRI is an added value for the future clinical implementation of this theranostic approach.

## Supplementary information

Supplementary information.
