# Supplementary material for: miR-216a-targeting theranostic nanoparticles promote proliferation of insulin-secreting cells in type 1 diabetes animal model
Source: Sci Rep. 2020 Mar 24;10:5302. doi: 10.1038/s41598-020-62269-4 (PMC7093482; doi:10.1038/s41598-020-62269-4)
Supplement: Supplementary file 1 — Supplementary information. [file 41598_2020_62269_MOESM1_ESM.pdf]

miR-216a-targeting theranostic nanoparticles promote proliferation of insulin-secreting cells in type 1 diabetes animal model

Authors: Ping Wang[1]*, Qiong Liu[2], Hongwei Zhao[3, 4], Jack Owen Bishop[1,5], Guoli Zhou[6], L. Karl Olson[7], Anna Moore[1]*

[1] Precision Health Program, Department of Radiology, College of Human Medicine, Michigan State University, East Lansing, Michigan 48823, USA.

[2] Department of Anatomy, Histology and Embryology, School of Basic Medical Science, Fudan University, Fudan University, Shanghai, 200032, China.

[3] Shanxi Medical University, Taiyuan, Shanxi, 030001, China.

[4] Department of Gynecologic Oncology, Shanxi Provincial Cancer Hospital, Taiyuan, Shanxi, 030013, China.

[5] Department of Neuroscience, College of Natural Science, Michigan State University, East Lansing, Michigan 48824, USA.

[6] Biomedical Research Informatics Core, Clinical & Translational Sciences Institute, Michigan State University, East Lansing, Michigan 48824, USA

[7] Department of Physiology, College of Natural Science, Michigan State University, East Lansing, Michigan 48824, USA.

*Address correspondence to: Ping Wang, Precision Health Program, Department of Radiology, Michigan State University, 766 Service Road, Rm. 2020, East Lansing, MI 48823, tel. (517)-353-3817, email: wangpin4@msu.edu, and Anna Moore, Precision Health Program, Department of Radiology, Michigan State University, 766 Service Road, Rm. 2022, East Lansing, MI 48823, tel. (517)-355-4091, email: moorea57@msu.edu

Supplemental material

Supplemental tables:

Supplemental table 1: Sequences of the miRNA mimics, inhibitors and scramble controls for nanodrugs synthesis and ISH

miRNA mimics, inhibitors, ISH probe	Sequences
miRNA-216a inhibitors	CACAGTTGCCAGCTGAGATT
negative control for inhibitors	GTGTAACACGTCTATACGCCCA
miRNA-216a mimics	UAAUCUCAGCUGGCAACUGUGA
scrambled control for mimics	UGGUUUACAUGUCGACUAAUU
miRNA-216a ISH probe	TCACAGTTGCCAGCTGAGATTA

Supplemental table 2: miRNA microarray data profiling of the islets from 3, 8 and 18-week-old NOD mice:
https://drive.google.com/file/d/1RSGUvNLcL4iwRXUyTeYsNfUcDZziRegy/view

Supplemental figures:

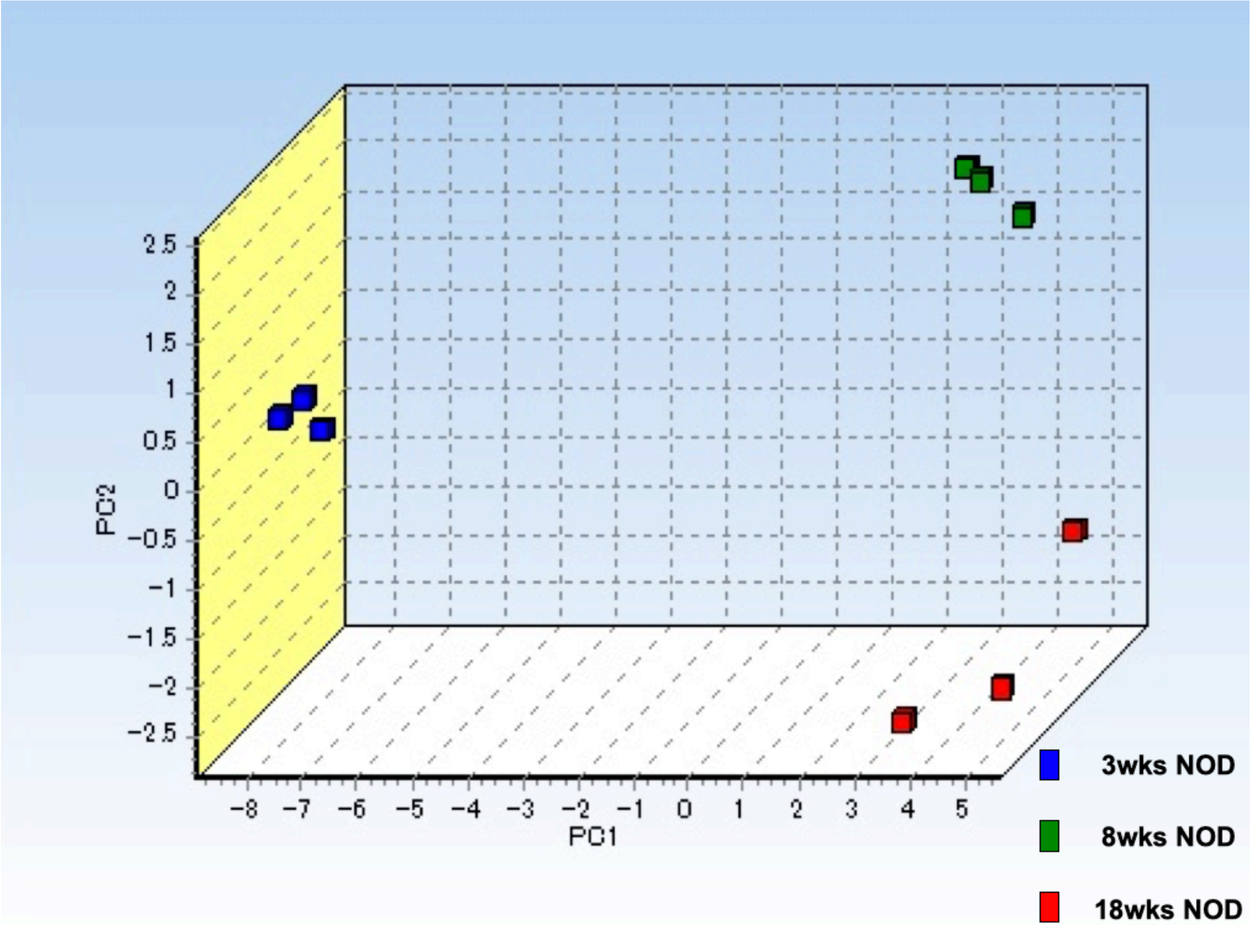

Supplemental Fig. 1. Principal components analysis (PCA) was used to reduce the dimensionality of multivariate data into a multi-dimensional space, allowing for clear visualization of the variation between the three microRNA microarray samples.

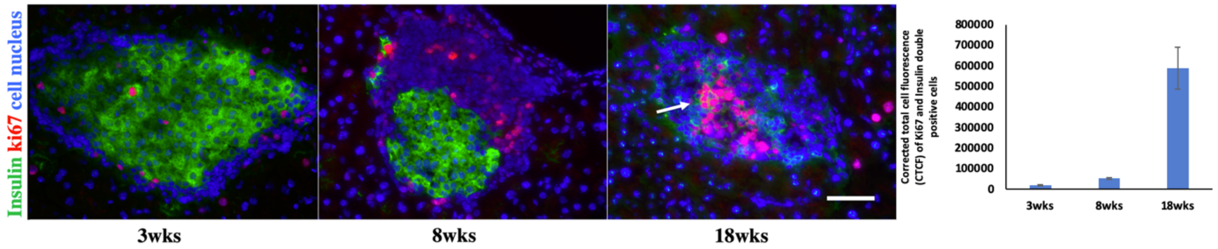

Supplemental Fig. 2. Immunostaining for insulin (green) and Ki67 (red) in pancreatic islets from 3-, 8- and 18-week old NOD mice (cell nucleus - blue, magnification bar = 50 μm); The arrow indicates double positive cells in damaged pancreatic islets of 18-week old NOD mice.

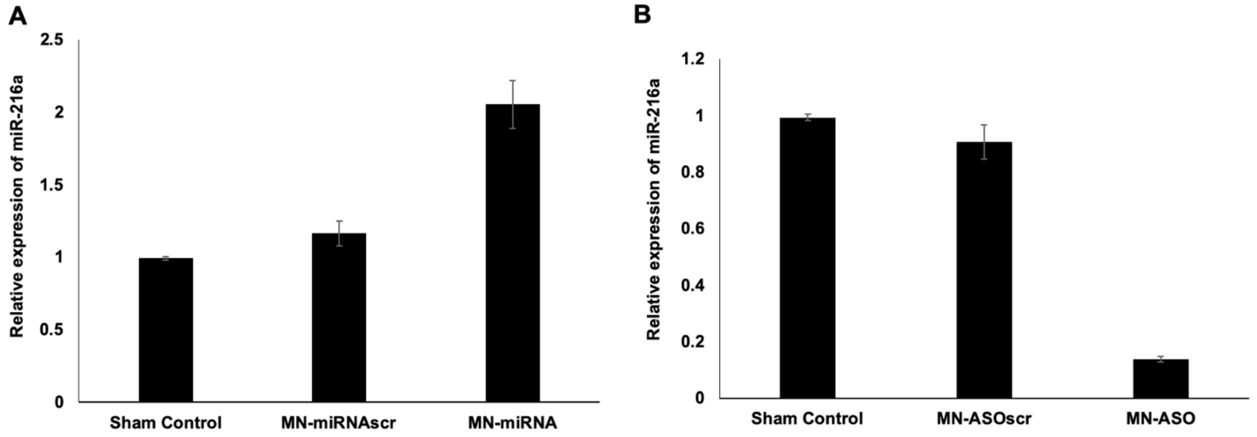

Supplemental Fig. 3. A. MN-miRNA mimic nanodrug increased miR-216a expression in beta-TC6 cells post incubation. B. MN-ASO inhibitor nanodrug significantly inhibited miR-216a in beta-TC6 cells compared to controls. These experiments were performed in triplicate.

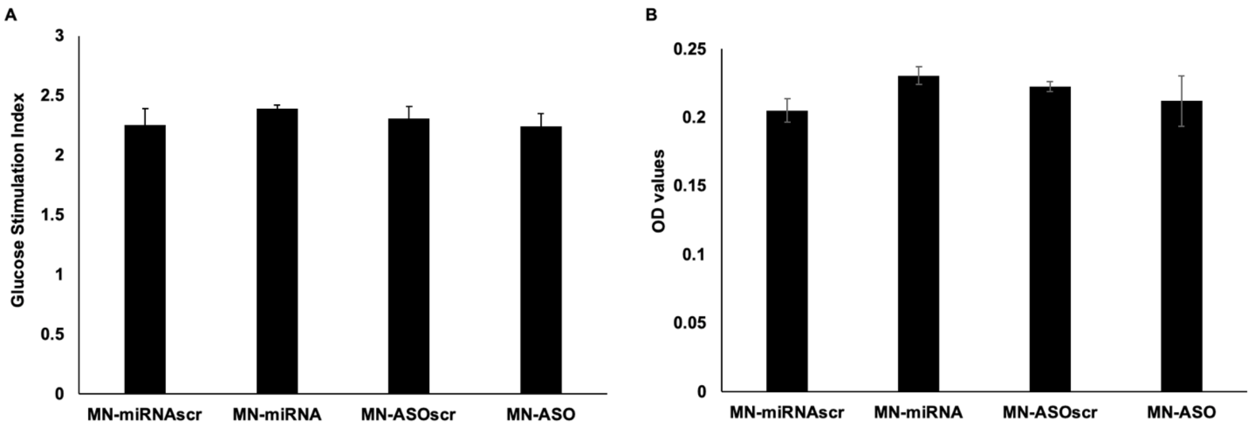

Supplemental Fig. 4. A. Glucose stimulation index of beta-TC6 cells was not significantly different in MN-miRNA-, MN-ASO-treated cells or controls. 4B: MTT assay revealed that incubation with MN-miRNA or MN-ASO nanodrugs did not cause significant toxicity in beta-TC6 cells.

Original imaging of the Western Blot gels:

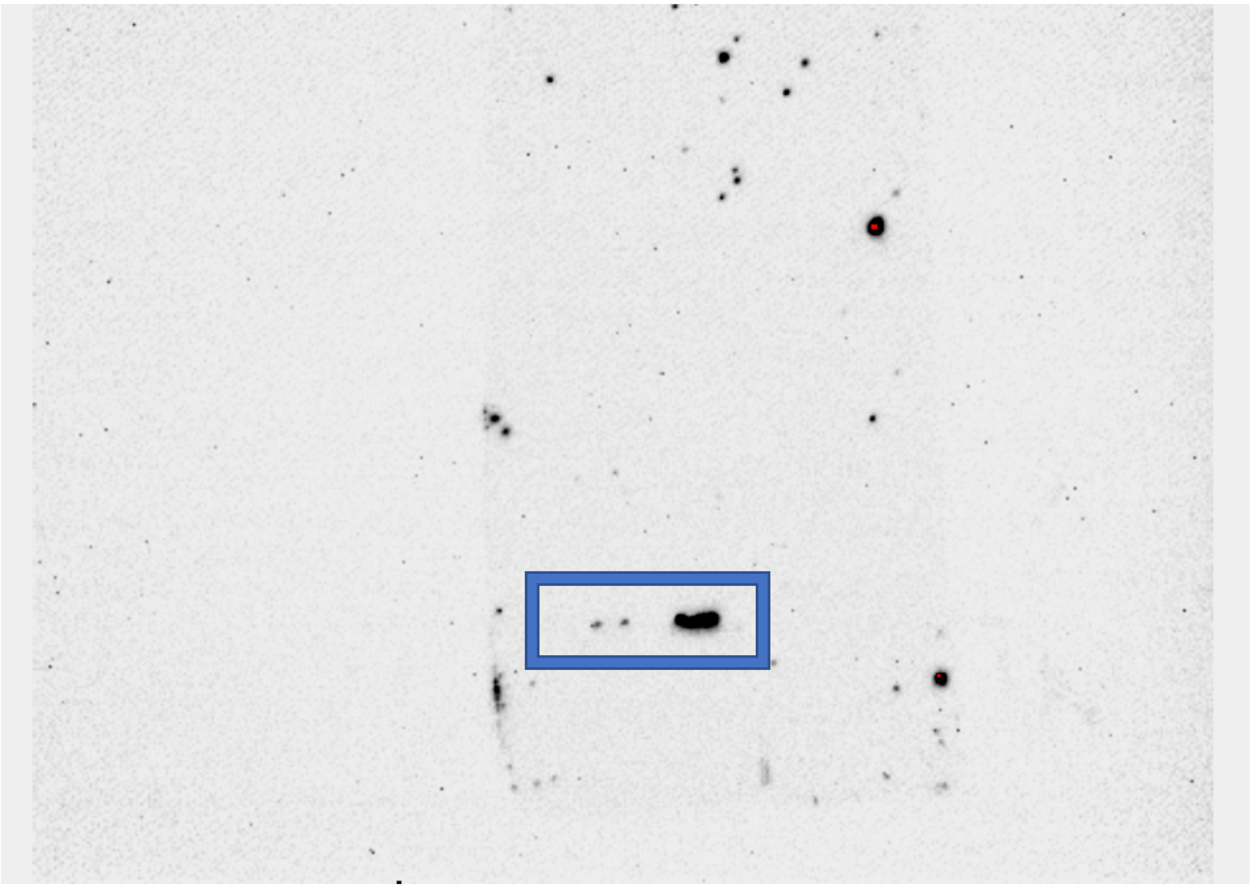

Full unedited gel/blot for Figure 4A top, PTEN expression

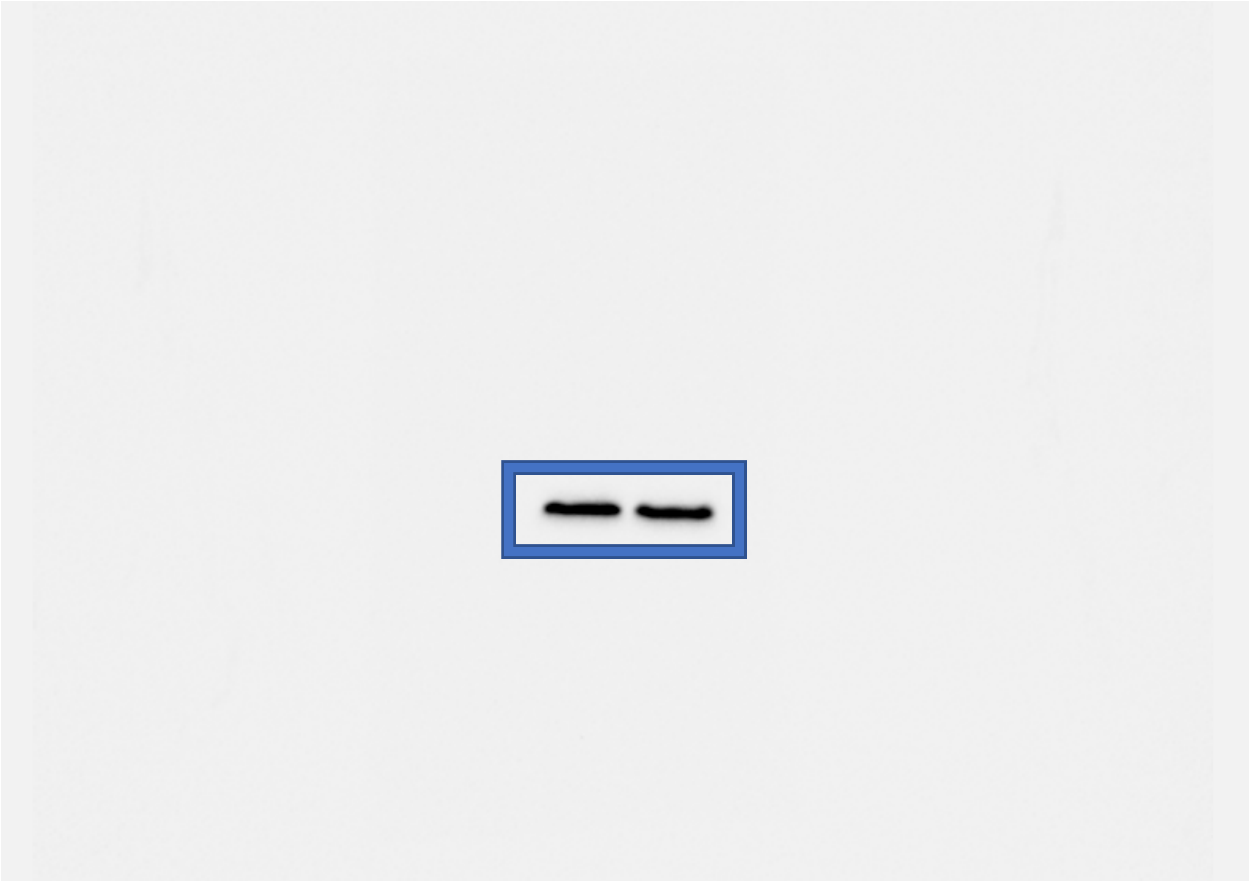

Full unedited gel/blot for Figure 4A top, actin expression

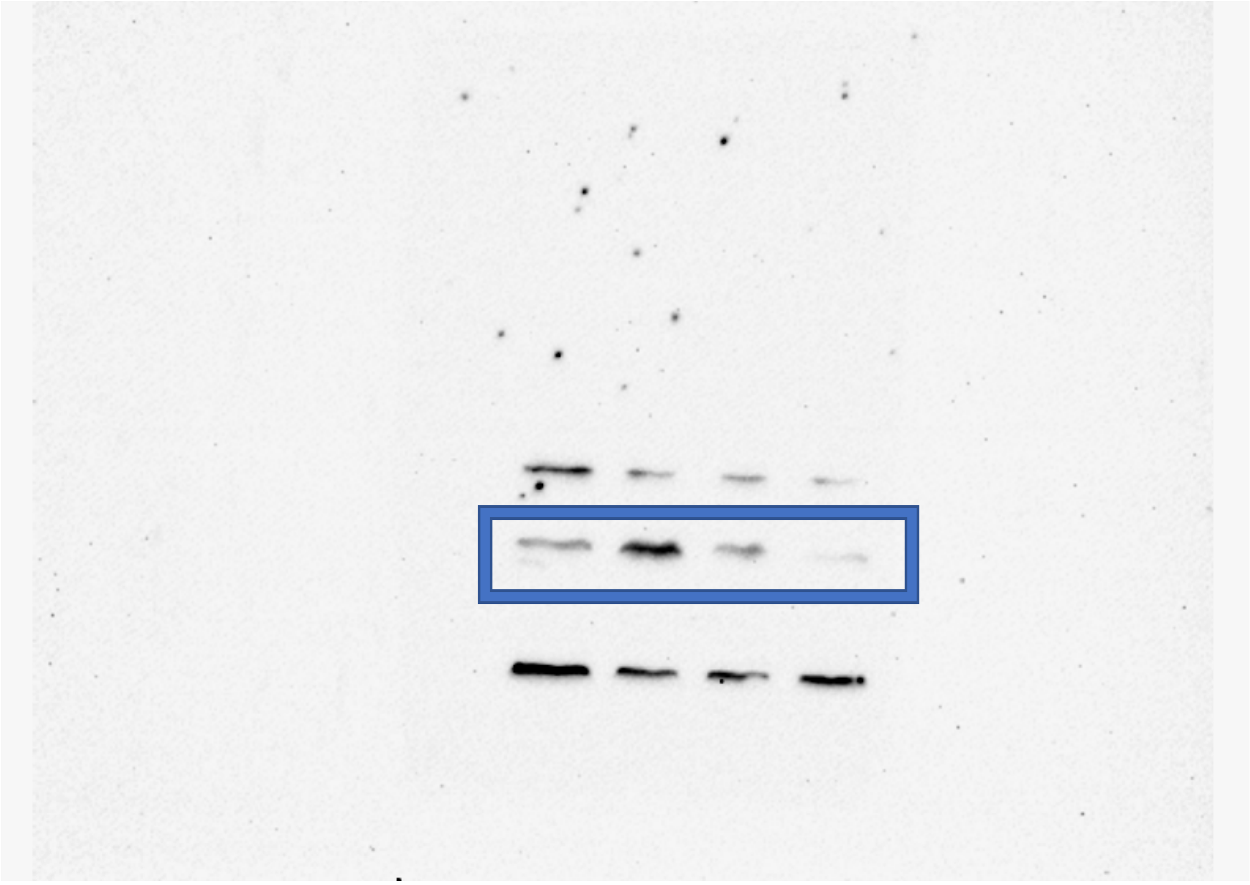

Full unedited gel/blot for Figure 4A bottom, PTEN expression

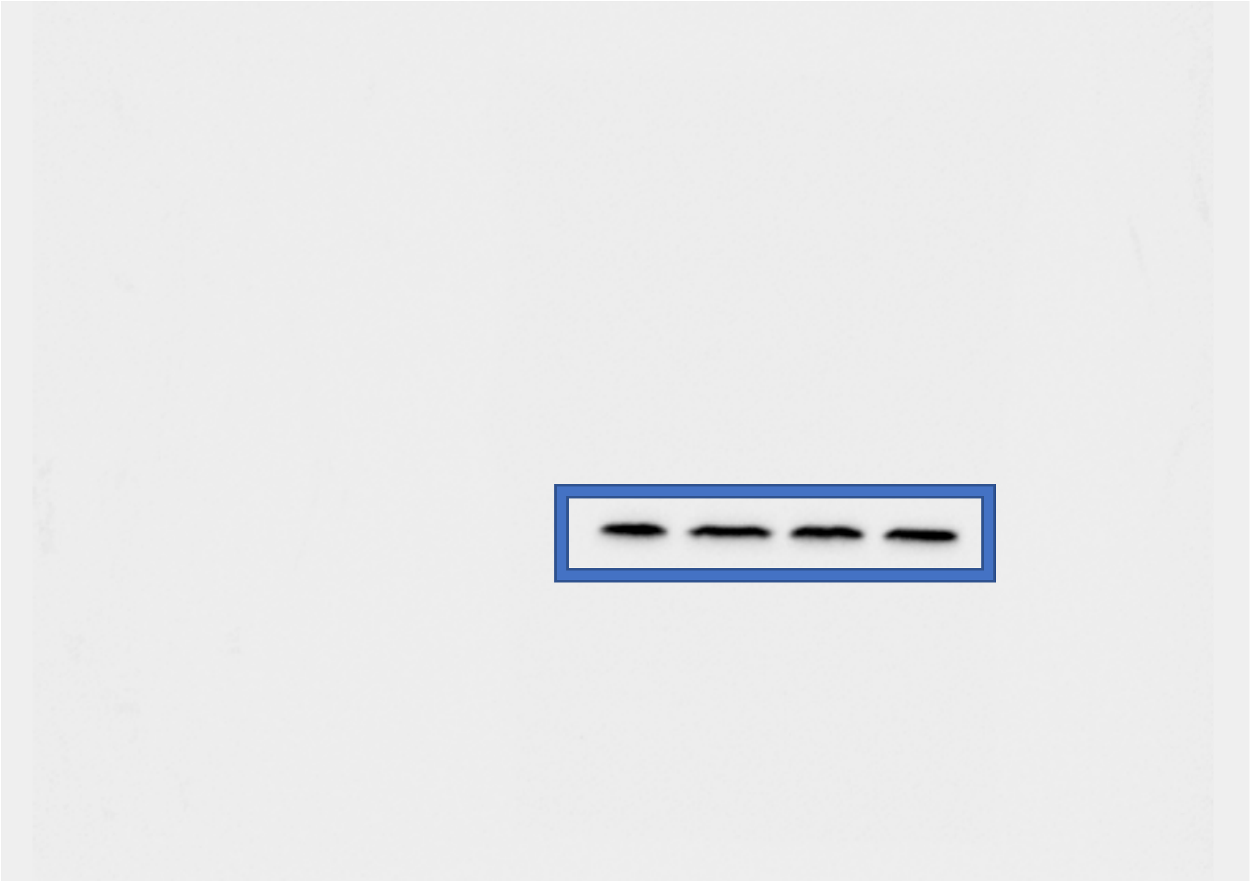

Full unedited gel/blot for Figure 4A bottom, actin expression

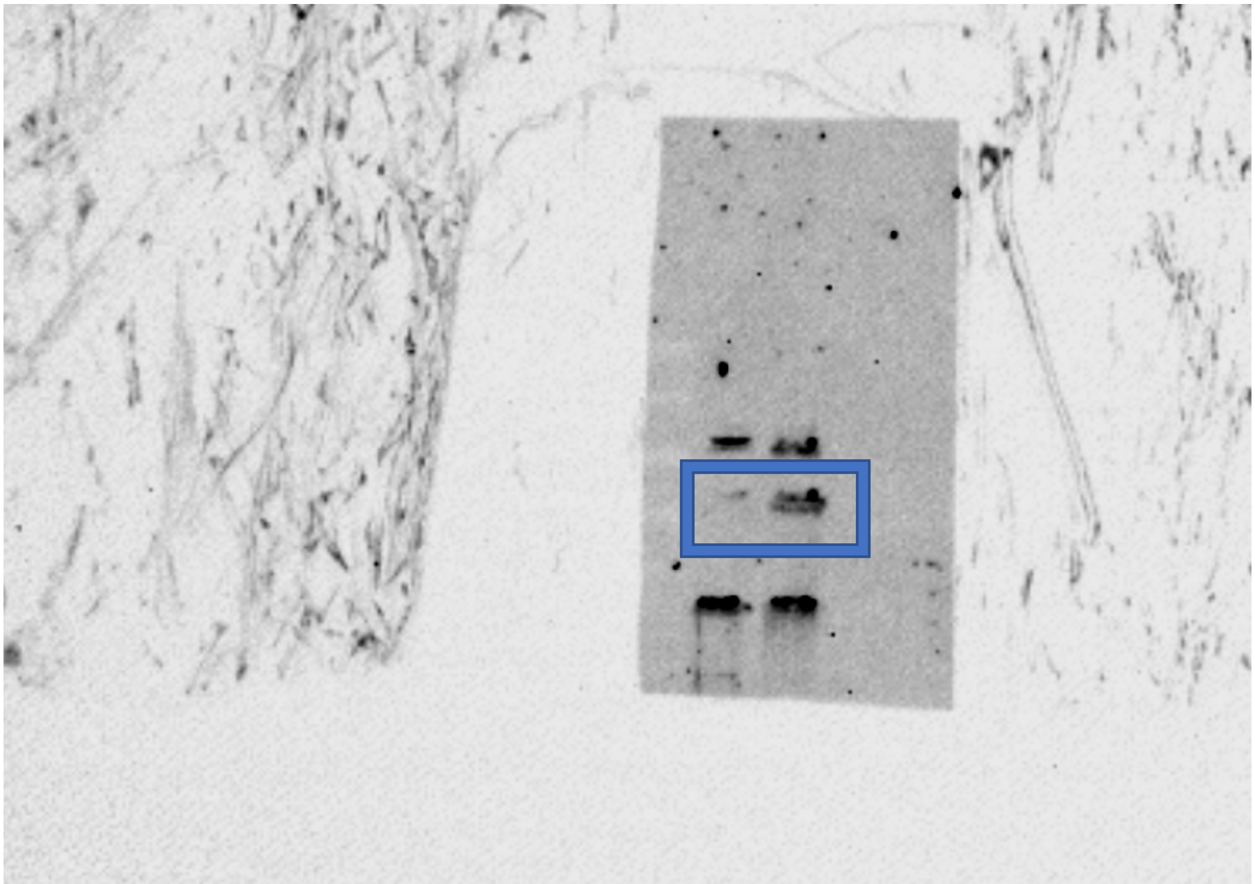

Full unedited gel/blot for Figure 4B top, PTEN expression

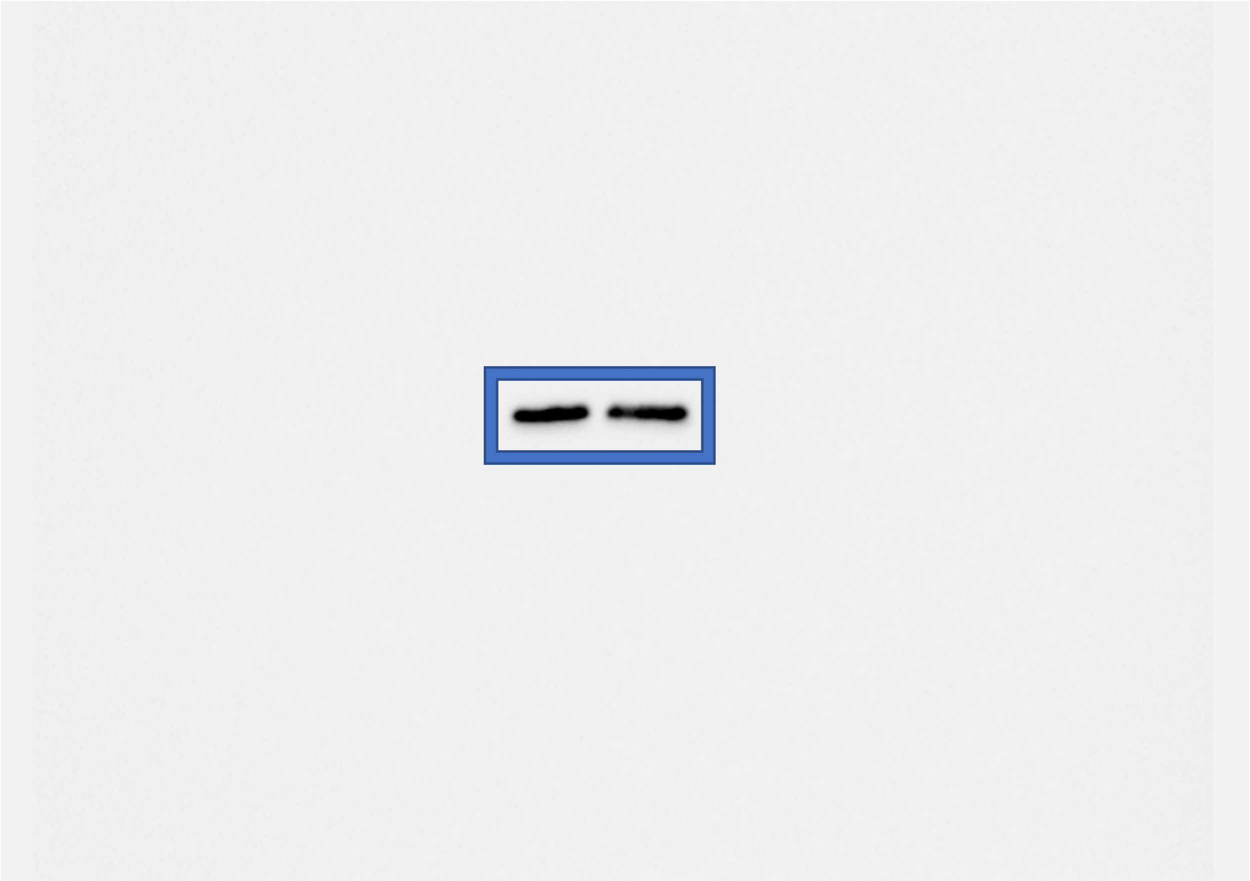

Full unedited gel/blot for Figure 4B top, actin expression

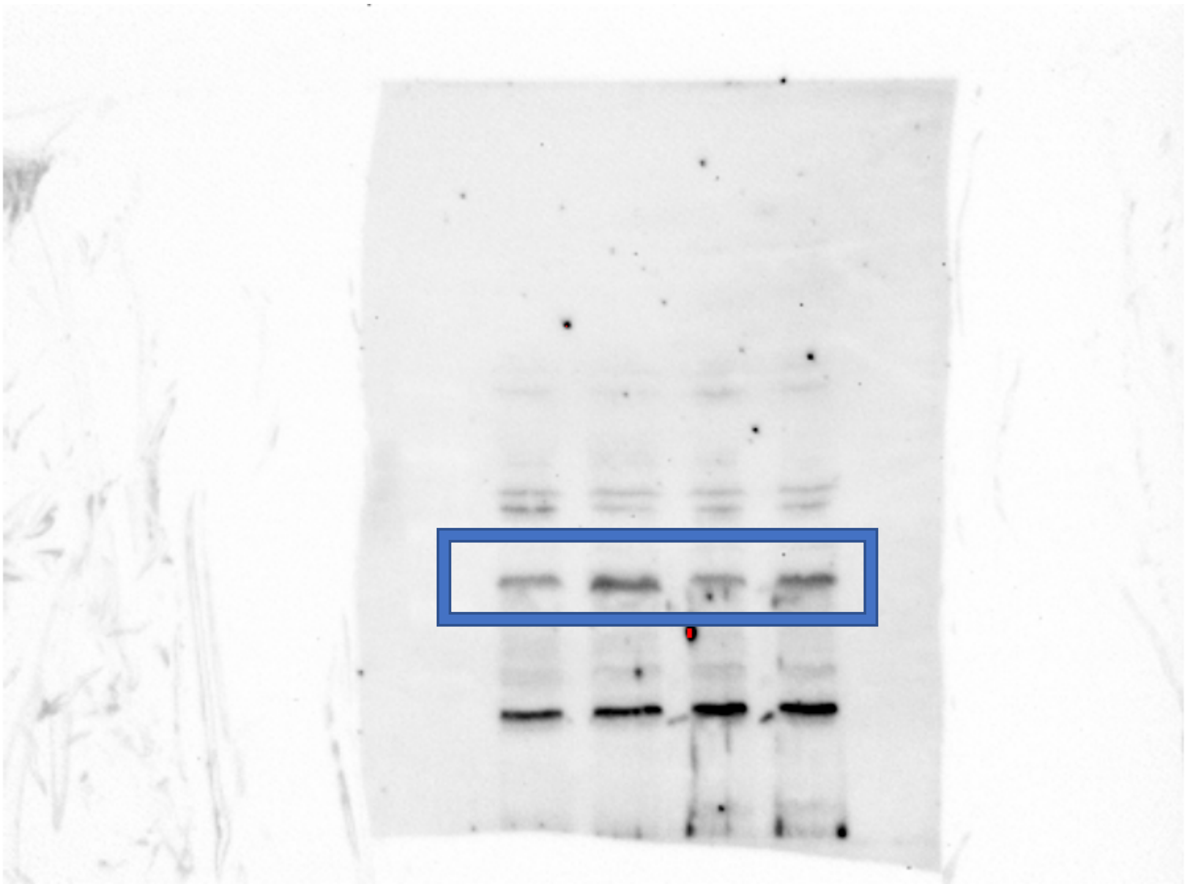

Full unedited gel/blot for Figure 4B bottom, PTEN expression

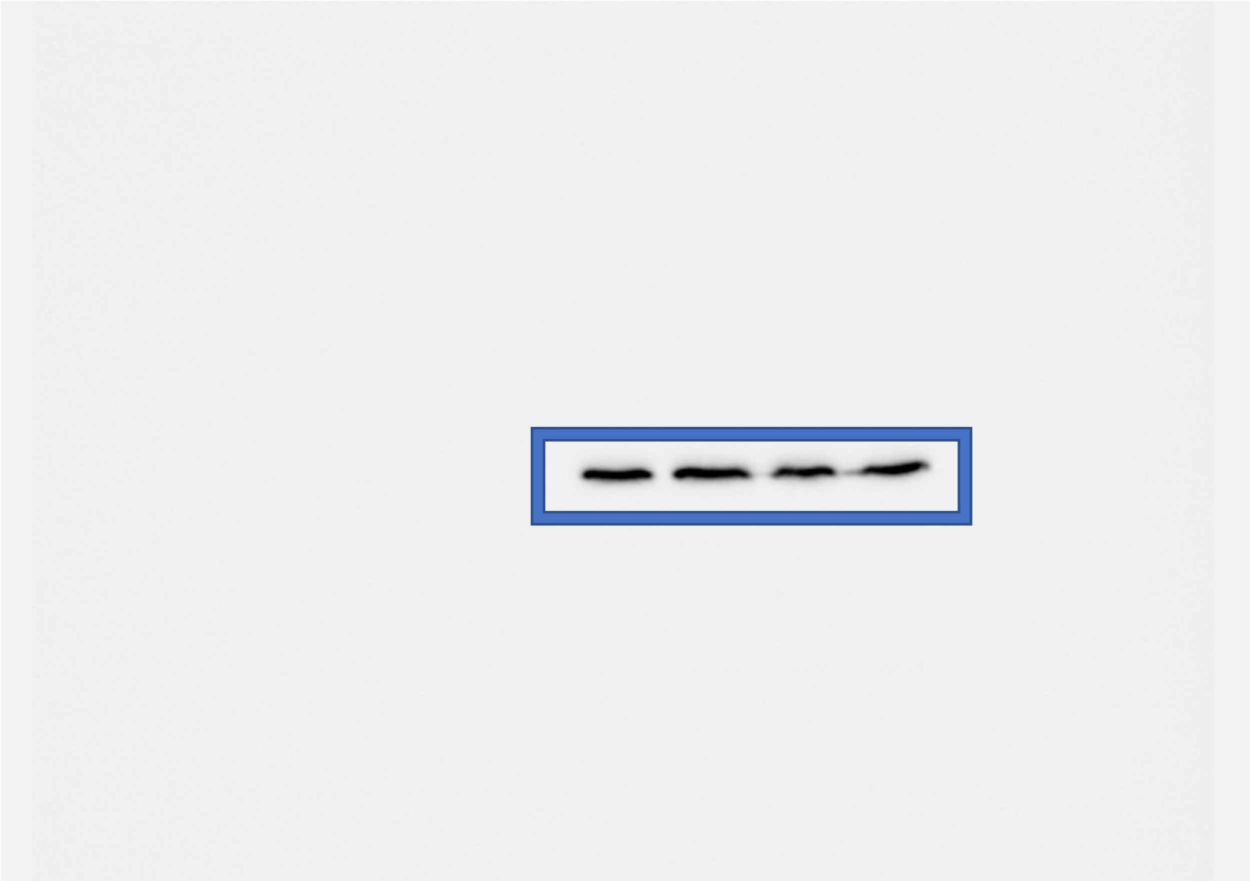

Full unedited gel/blot for Figure 4B bottom, actin expression
